# The long-term general practice healthcare of women with a history of gestational diabetes: A Scoping Review Protocol

**DOI:** 10.12688/hrbopenres.14022.2

**Published:** 2025-04-10

**Authors:** James O'Flynn, Rita McMorrow, Tony Foley, Rita Forde, Sheena McHugh, Christine Newman, Aisling A. Jennings

**Affiliations:** 1 Department of General Practice, University College Cork School of Medicine, Cork, T12 XF62, Ireland; 2 The Department of General Practice, The University of Melbourne, Melbourne, Victoria, Australia; 3 School of Nursing and Midwifery, University College Cork, Cork, T12 XF62, Ireland; 4 School of Public Health, University College Cork, Cork, T12 XF62, Ireland; 5 School of Medicine, College of Nursing, Midwifery and Health Science, University of Galway, Galway, H91 TK33, Ireland; 6 Diabetes Collaborative Clinical Trial Network, University of Galway, Galway, H91 TK33, Ireland

**Keywords:** Gestational Diabetes Mellitus, Follow-Up, General Practice, Primary Care, Scoping Review, Type 2 Diabetes Mellitus

## Abstract

**Introduction:**

Gestational Diabetes Mellitus (GDM) is a hyperglycaemic condition diagnosed during pregnancy. GDM is strongly associated with future development of type 2 diabetes and cardiovascular disease. Lifestyle and pharmacological interventions can reduce the risk of developing type 2 diabetes. General practice is the recommended setting for long-term follow-up of women with a history of GDM. However, rates of follow-up are suboptimal. The evidence around long-term general practice healthcare for women with a history of GDM has not previously been reviewed.

**Aims:**

The aim of this scoping review is to explore the current evidence base for the long-term care of women with a history of GDM in general practice.

**Study Design:**

The study described by this protocol is a scoping review. The study design was informed by Joanna Briggs Institute methodology.

**Methods:**

Empirical qualitative and quantitative research studies published since 2014 will be identified from a search of the following databases: MEDLINE (Ovid), EMBASE (Elsevier), CINAHL, PsycINFO, Academic Search Complete and SocIndex. The review will identify key characteristics of the literature. Framework analysis will be used to map the findings against the Chronic Care Model, a primary care-based framework that sets out the core components for optimal long-term healthcare.

**Results:**

A numerical descriptive summary (using frequencies) will describe the overall extent of literature, and the range and distribution of its component parts, including the geographical and economic settings, research methods, interventions, outcomes and findings. The qualitative analysis will map interventions and descriptions of care to components of the chronic care model. Research gaps will be reported, and research needs and priorities will be suggested.

**Conclusion:**

The findings of this scoping review will have the potential to inform future research efforts in the area.

**Registration:**

This protocol has been registered in Open Science Framework ( https://osf.io/bz2vh).

## Introduction

Gestational Diabetes Mellitus (GDM) is a hyperglycaemic condition with onset or first recognition during pregnancy ^1^. The prevalence of GDM is estimated to be at 14% of pregnancies globally using the International Association of Diabetes in Pregnancy Study Group diagnostic criteria ^2^. The prevalence is increasing, which has been linked to advancing maternal age and rising obesity rates ^3, 4^. GDM is associated with pregnancy complications for both mother and foetus, such as macrosomia and pre-eclampsia ^5– 7^. Pregnancy and perinatal GDM management is associated with increased economic costs ^8– 10^. Rates of GDM in subsequent pregnancies are between 30% and 84% ^11^.

GDM further presents enduring health risks for women after pregnancy. Women who experience GDM have an approximately tenfold risk of later developing type 2 diabetes ^12, 13^. It has been estimated that up to one in three women with type 2 diabetes experienced a pregnancy which was complicated by GDM ^14^. Even in the absence of diabetes, GDM is associated with increased cardiovascular risks, including ischaemic heart disease, heart failure, thromboembolic disease and stroke ^15, 16^. Emerging research suggests potential links between GDM and increased rates of other conditions, including hepatic steatosis, renal disease and cancers of the breast, reproductive tract and thyroid ^17– 21^. A greater susceptibility to obesity and metabolic syndrome has also been identified among children of women with a history of GDM ^22, 23^. Consequently, GDM carries the potential for lifelong health consequences for the mother as well as intergenerational health implications ^24, 25^.

To date, research relating to the management of GDM has primarily focused on pregnancy and early postpartum care after a diagnosis of GDM. Interventions aimed at preventing type 2 diabetes after a GDM diagnosis include lifestyle, education and medication ^26^. A recent meta-analysis found that the hazard ratio of type 2 diabetes was significantly improved by interventions, while the relative risk did not achieve significance ^27^. This may occur if the effect of interventions is time-dependent, and may suggest that their effect may more readily delay than outright prevent the onset of type 2 diabetes. Evidence syntheses indicate that interventions to support healthy diet and physical activity for women with a history of GDM may result in improvements in glycaemic measures and adiposity, with lower rates of subsequent type 2 diabetes ^28– 32^. However, these results have not consistently achieved statistical significance. Studies report multiple barriers for women engaging with lifestyle interventions, including maternal tiredness, competing occupational and family demands, and personal risk perception ^33, 34^. Educational interventions targeting postpartum women with a history of GDM have not predominantly been studied in isolation, and have been used primarily as an adjunct to or vehicle for diet and physical activity interventions ^35– 39^. Limited pharmacological studies have primarily examined glucose-lowering therapies. Meta-analyses inconsistently identify modest reductions in hyperglycaemia and progression to type 2 diabetes, though large scale and long-term data is lacking ^26, 27, 40, 41^. Deficits in the reporting of socioeconomic factors in the evidence for prevention of type 2 diabetes for women with a history of GDM has previously been demonstrated ^42^. Despite the limitations of the evidence for optimal long-term interventions for this population, economic analyses have found that interventions designed to prevent the development of type 2 diabetes for women with a history of GDM appear cost-effective ^43, 44^. Data regarding the role for preconception care aimed at reducing the risk of GDM recurrence are also lacking ^45– 47^. In spite of this, clinical practice guidelines frequently identify the importance of family planning and contraception for women with a history of GDM ^48^. To the best of our knowledge, the effects of interventions on the prevention of other diseases associated with GDM, such as cardiovascular disease, have not been studied ^49^.

Due to the long-term risks associated with GDM and the opportunities for subsequent disease prevention, clinical practice guidelines recommend regular long-term follow-up healthcare. This may include lifelong regular screening for early diagnosis of diabetes or cardiovascular disease, and ongoing support for maintenance of positive health behaviours ^41, 50– 52^. Despite this, evidence suggests that rates of long-term follow-up of women who have been diagnosed with GDM are suboptimal, with less than half of women receiving follow-up diabetes screening after 1 year postpartum ^53– 55^. General practice (GP) has been identified as an appropriate setting for long-term follow-up care of women with a history of GDM ^24, 56^. GPs can provide direct support and can connect women with parallel services, including Diabetes Prevention Programmes ^57, 58^. A descriptive cross-sectional survey conducted in Canada found that three quarters of women with a history of GDM appear satisfied to transition to primary care-based settings after pregnancy ^59, 60^. However, fragmentation of care, deficits in interprofessional communication and uncertainty regarding professional roles and responsibilities can impact on the long-term care provided to these women ^59, 61, 62^. Optimal models of care and factors influencing their delivery have not been established.

To our knowledge there have been no systematic attempts to provide an overview of the available research evidence on the long-term general practice-based healthcare of women with a history of GDM. Therefore, a comprehensive understanding of long-term general practice healthcare for women with a history of GDM is required if the rates and quality of follow-up are to be improved. The aim of this scoping review is to explore the current evidence base for the long-term care of women with a history of GDM in general practice.

## Objectives

What are the extent, nature and key characteristics of research exploring long-term GP-based healthcare for women with a history of GDM, including:

- The experiences and perspectives of women with a history of GDM and their GPs
- Interventions studied in a GP setting, with particular focus on the range of interventions, the reporting of interventions and the reporting of socioeconomic characteristics of study populations
- Outcomes studied, with particular focus on physical outcomes to include the prevention of type 2 diabetes and cardiovascular disease and psychological outcomes
- What areas of the healthcare system are well-researched, and where are there research gaps, employing the Chronic Care Model as a guiding framework

## Methods

The JBI Manual for Evidence Synthesis builds on previous scoping review methodological frameworks, most notably Arksey and O’Malley, and will guide this scoping review protocol ^63, 64^. Guidance will also be taken from the Preferred Reporting Items for Systematic Reviews and Meta-Analyses (PRISMA) protocol framework ^65^.

### Eligibility criteria

Studies will be selected according to the criteria outlined in Table 1. Explanation of the criteria follows.

**Table 1.** Eligibility criteria for studies in the scoping review.

Category	Inclusion Criteria	Exclusion Criteria
Population	Non-pregnant adults with a previous history of GDM, diagnosed using established criteria	People living with other forms of diabetes Offspring of women with a history of GDM
Concept	Research examining any characteristic of healthcare occurring after initial post-partum glycaemia screening	Studies examining initial post-partum glycaemia screening only Studies that do not describe or evaluate healthcare following a diagnosis of GDM
Context	General Practice in any geographical or economic setting	Studies not based in general practice, including healthcare delivered in the community, home, hospital or through the internet Healthcare services led by specialists other than general practice (e.g. dietetics, physiotherapy, midwifery)
Language	Any	None
Types of Evidence Sources	Peer reviewed Empirical research including: Qualitative research Quantitative research Mixed-methods research Economic analyses	Case reports Case series Opinion pieces Evidence syntheses Position papers Clinical practice guidelines Policy documents Dissertations or theses

***Population***

We will include studies examining general practice healthcare of non-pregnant adults with a previous history of GDM. Heterogenous screening approaches and diagnostic criteria are used for GDM, with variations of oral glucose tolerance testing and the associated diagnostic thresholds adopted by different clinical practice guidelines ^66^. Studies including any internationally accepted criteria for the diagnosis of GDM will be accepted. Studies involving adults with prediabetes after a GDM diagnosis, established type 2 diabetes after a GDM diagnosis, pre-existing diabetes, or other forms of diabetes will be excluded. For the purposes of this review, due to the absence of clinical guideline recommendations for structured long-term follow-up of children of women with a history of GDM, studies involving children will be excluded unless maternal general practice healthcare is also addressed in the study.

***Concept***

We will review research studies which investigate long-term healthcare of women with a history of GDM in general practice. This may include general practice-based research on interventions for prevention of diseases including GDM recurrence, type 2 diabetes or cardiovascular disease following GDM, as well as processes for screening or diagnosis of such conditions. We will also include studies examining factors that increase or decrease rates of the delivery and uptake of GP healthcare. We will include studies examining interactions between GPs and women with a history of GDM, or GPs and other health professionals regarding the care of these women. We will include studies exploring stakeholder perspectives of care in general practice. Stakeholders may include GPs, GP staff or women with a history of GDM receiving GP care. Educational interventions targeting GPs and GP staff will be included. Studies of care pathways or care programmes and economic analyses of long-term general practice healthcare for women with a history of GDM will be included. Studies that compare long-term general practice healthcare for women with a history of GDM with other forms of healthcare will be included. We will exclude studies examining early post-partum follow-up care. Long-term healthcare for women with a history of GDM will be defined for this study as healthcare provided beyond the early postpartum period.

***Context***

Studies included in this review will involve general practice settings, defined according to the WONCA definition ^67^. This will exclude community- or home-based interventions aimed at promoting self-management only. This will also exclude evidence pertaining to hospital or specialist care only. Interventional studies carried out through research institutions or universities will be excluded if they are not delivered within a clinical general practice setting. Other forms of primary care, such as dietitian-led care, will not be included in this review. Studies occurring in any geographical and economic context will be considered, provided the care occurs within a general practice setting.

***Language***

No language restrictions will be applied. Non-English language studies will be translated using freely available proprietary translation software programmes. Initial translation will be carried out using Google Translate, with unclear translations being cross-checked with CUBITT translation ^68, 69^.

***Types of evidence sources***

This scoping review will include published empirical qualitative, quantitative or mixed-methods studies. Economic analyses will be included. Primary sources cited by evidence syntheses arising from the search process will be included, but the evidence syntheses themselves will not be included. Grey literature will not be included. Case reports, case series and opinion pieces will not be included. We will exclude clinical practice guidelines, policy documents and the position papers of scientific bodies. We will exclude dissertations and PhD theses.

### Search strategy

A health information specialist with experience in scoping reviews was consulted in the design of the search strategy. The three-step search approach recommended by JBI will be used ^64^. After an initial search to assess relevant keywords and indexing terms, the search strategy was developed using medical subject headings (MeSH) and natural language text words or phrases related to the core concepts of GDM, GP and a Long-Term timeframe. We will search MEDLINE (Ovid), EMBASE (Elsevier), CINAHL, PsycINFO, Academic Search Complete and SocIndex. The search terms for MEDLINE (Ovid) are provided in Table 2. To accommodate the rapidly evolving healthcare landscape, and to permit permeation of the practice-changing findings of the Hyperglycemia and Adverse Pregnancy Outcomes study 2008, the search years will be limited to the past 10 years (2014 or later) ^70^. To ensure a comprehensive search of published literature, reference lists and studies citing the accepted papers will be scanned. The reference lists of systematic reviews identified in the search will also be scanned.

**Table 2.** Search (MEDLINE (Ovid)).

	Query
1	("Gestational Diabetes" or GDM or "Pregnancy-Induced Diabetes" or (Pregnancy and (Diabetes or Hyperglycaemia or Dysglycaemia or "Glucose intolerance" or "Impaired glucose tolerance"))).ti. or ("Gestational Diabetes" or GDM or "Pregnancy- Induced Diabetes" or (Pregnancy and (Diabetes or Hyperglycaemia or Dysglycaemia or "Glucose intolerance" or "Impaired glucose tolerance"))).ab.
2	(Long-Term or "Long Term" or Follow-up or "Follow up" or "longitudinal" or "Chronic disease management" or "late complications" or "delayed effects").ti. or (Long-Term or "Long Term" or Follow-up or "Follow up" or "longitudinal" or "Chronic disease management" or "late complications" or "delayed effects").ab.
3	("General Practice" or "General Practitioners" or "GP" or "Family medicine" or "Family Physicians" or "FP" or "Primary Care" or "Primary Healthcare" or "PCP").ti.or ("General Practice" or "General Practitioners" or "GP" or "Family medicine" or "Family Physicians" or "FP" or "Primary Care" or "Primary Healthcare" or "PCP").ab.
4	Diabetes, Gestational.sh.
5	(Follow-up Studies or Chronic Disease).sh.
6	(General Practice or General Practitioners or Primary Health Care or Physicians, Primary Care or Family Practice or Physicians, Family).sh.
7	1 or 4
8	2 or 5
9	3 or 6
10	7 and 8 and 9

### Source of evidence selection

Results will be managed with the systematic review tool, Covidence ( www.covidence.org). A pilot selection process will be conducted on 100 papers to refine the process, according to JBI recommendations. Full screening will proceed once agreement is at least 75%. After the full search is completed, two reviewers will independently conduct screening according to the eligibility criteria (RMcM, JOF). Initial title and abstract screening will be followed by full text screening of apparently eligible articles. Disagreements will be resolved by consensus or by a third reviewer (AJ). If additional information is required from authors to confirm eligibility status of a source, the corresponding author will be contacted by e-mail. Reasons for exclusion of full text sources will be recorded. The numbers of citations identified, duplicates removed, additions via alternative sources, records screened, and full text reviews will be reported. Inter-rater agreement for the screening of search results will be reported. The report of the selection process will be accompanied by a visual flowchart according to the PRISMA extension for scoping reviews ^71^.

### Data extraction and analysis

***Descriptive numerical summary***

For the descriptive numerical summary of studies included in this review, data extraction will be developed and piloted to record the key information from each source ^63^. The process is intended to map key features of the literature, such as study design, aims, methods, intervention type (if applicable) and key findings ^63, 72^. The factors for inclusion on the initial data extraction form are included in Table 3. The items chosen are suggested in the JBI methodology, and minor additions are included here. We will record studies’ reporting of population characteristics according to the PROGRESS framework. This framework is used to identify key population factors influencing equity of outcomes, which are place of residence, race, occupation, gender, religion, education, socioeconomic status and social capital ^73^. Reporting of these items permits assessment of inequity within a study ^74^. The presence of an equity assessment within research is desired by policymakers ^75^. Intervention descriptions and outcome details will be extracted from interventional studies. We will record the reporting of interventions in studies according to the Template for Intervention Description and Replication (TIDieR), a 12-item tool designed to improve the completeness of reporting of interventions ^76^. Outcomes will be categorised according to the core outcome sets designed for the reporting of interventional studies in this population ^77, 78^. Psychological or emotional outcomes will be included in addition to the core outcome set items. This activity will be refined iteratively at the review stage by one author (JOF) applying the initial data extraction form to five to ten papers. Two authors (CN, JOF) will discuss and adjust until the forms are used consistently, and the data emerging contribute to answering the research question ^72^.

**Table 3.** Factors for inclusion on the initial data extraction form.

Item	Data for Extraction
Author	First author
Year of Publication	Year
Setting	Country / Countries
	Economic developmental category (according to the World Bank’s Gross National Income per capita)
Aims / Purpose	Self-identified aim
Methodology	Methodological design
Population (if applicable)	Sample Size
	Subgroup items reported (PROGRESS)
	Duration of follow-up (if applicable)
Intervention (if applicable)	Intervention description (TIDieR)
	Duration of intervention
Outcome (if applicable)	Primary Outcomes
	Secondary Outcomes
Key Findings	Key Findings
	PROGRESS subgroup differences
Research Recommendations	Author Recommendations for Future Research

Analysis of the extracted data will involve the use of descriptive statistics to provide an overview of the included studies according to their key characteristics. Frequencies will be the predominant statistical method used. For evaluations of interventional studies, the frequencies of outcomes reported in relation to the core outcome set will also be described.

***Qualitative mapping***

The qualitative arm of the review will systematically categorise and map findings from included studies. A framework synthesis will be conducted ^64, 79, 80^. Framework methodologies use a highly structured, transparent approach to mapping data in a manner appropriate to scoping reviews ^64, 80^. The components of the Chronic Care Model will be used as a ‘Best Fit' *a priori* framework based methods described by Carroll and colleagues ^81, 82^. The Chronic Care Model is a primary care-based theoretical model which sets out the core components of a system of high-quality chronic disease management ^83– 85^. These components are the community linkage, the self-management support, organisation of healthcare delivery system, delivery system design, decision support and clinical information systems ^83, 84^. Implementation of these components is associated with benefits for people with a history of type 2 diabetes ^85– 89^. Adoption of the Chronic Care Model in the design of approaches to the diabetes management has been recommended by the American Diabetes Association ^90^. No pre-existing theoretical frameworks specifically designed for understanding the care of women with a history of GDM were identified by the authors.

The qualitative analysis and mapping will involve four authors (RF, AJ, RM, JOF). One researcher (JOF) will extract the relevant text data from all included studies to word processing software (Microsoft Word). The units of analysis will be the intervention descriptions, verbatim quotations from participants in primary studies, and the qualitative findings of the studies. NVivo software ( www.lumivero.com) will be used for the coding process. Two authors (RF, JOF) will conduct a pilot coding process on two papers. This will involve line-by-line deductive coding of the extracted data against the *a priori* framework ^81, 82^. Existing definitions of the Chronic Care Model components will be used to guide the coding and categorisation. Team discussion will be used to specify subcategories that will further contribute to characterising the distribution of data in this subject area. Extracted data not accommodated by the framework will be inductively coded ^82, 91^. The pilot coding will be subject to discussion among the review team regarding the coding rationale and its alignment with the research objective. The research team will meet to discuss the coding after this initial pilot. One author (JOF) will then independently conduct coding of the full dataset. Data charting involves rearranging the coded data according to the framework components ^79, 80^. Coded data will be charted, and inductive codes not falling within the framework will be gathered for consideration of significant new concepts ^81^. The research team will meet regularly and collectively interpret and map charted and inductively coded data, with particular focus on implications for the research question ^79– 81^. A final “map” that describes the evidence will then be produced in the form of a narrative summary.

***Gaps, needs and priorities***

Research gaps are areas where insufficient evidence prevents the formation of conclusions for a given research question ^92^. Research needs are gaps that may inhibit stakeholder decision-making, while research priorities are gaps prioritised due to resource constraints ^92^. Systematic methodologies for identifying gaps may also incorporate assessments of the quality of evidence beyond the scope of a scoping review ^64, 93^. In this case, gaps will be identified only from areas of absence or sparsity of findings in the descriptive numerical summary, outcome reporting and qualitative map ^93^. Gaps will be reported according to the pertinent areas of inadequacy using the population, intervention, comparison, outcome and setting framework (PICOS) ^93, 94^. Stakeholder consultation is an approach frequently used to identifying needs and priorities ^92, 94^. Potential research needs and priorities will be proposed from discussion of the review findings among the current multidisciplinary research team, comprised of individuals from several important stakeholder groups on the topic: general practice, public health, health services research, nursing and endocrinology. Research recommendations stated within the reports of included studies will be identified through the data extraction process, as included in Table 3. These recommendations will further inform the proposal of potential research needs and priorities.

### Reporting

The PRISMA extension for Scoping Reviews (PRISMA-ScR) will be used to guide the report ^71^.

### Dissemination plan

The intended audiences for this study will be researchers, health care professionals and policymakers who are involved in contributing to the structure of healthcare for women with a history of GDM, as well as the women themselves. This study will be submitted for publication in a peer-reviewed scientific journal and for scientific conference presentation. A plain English summary will be produced for women with a history of GDM.

## Discussion

This review will synthesise research evidence on the long-term healthcare for women with a history of GDM in general practice. This corresponds with recent interest in a longitudinal perspective on GDM ^24^. The methods detailed in this study protocol will provide transparency to the study’s findings. Incorporating both qualitative and quantitative papers, and the use of a well-established theoretical model, the chronic care model, will strengthen the findings of the paper. However, because no previous theoretical models have been applied to the healthcare of women with a history of GDM, the study may identify a need for a new, tailored model to understand long-term healthcare for women with a history of GDM. The use of a multidisciplinary research team with expertise from General Practice, Endocrinology, Nursing and Public Health will further strengthen the paper’s methodology and the interpretation of its results. Future research studies will be guided towards research gaps identified in this study.

## Conclusion

This paper outlines a study protocol for a scoping review on the topic of GP healthcare for women with a history of GDM. The review will provide a synthesis of the current research evidence on the topic, and research recommendations regarding long-term follow-up of women with GDM in general practice.

## Ethics and consent

Ethical approval and consent were not required.

## Data Availability

No data are associated with this article.

## References

[1] American Diabetes Association: Diagnosis and classification of diabetes mellitus. *Diabetes Care.* 2009;32 Suppl 1(Suppl 1):S62–7. 10.2337/dc09-S062 19118289 PMC2613584

[2] Wang H Li N Chivese T : IDF diabetes atlas: estimation of global and regional Gestational Diabetes Mellitus prevalence for 2021 by International Association of Diabetes in Pregnancy Study Group’s criteria. *Diabetes Res Clin Pract.* 2022;183: 109050. 10.1016/j.diabres.2021.109050 34883186

[3] Gortazar L Flores-Le Roux JA Benaiges D : Trends in prevalence of gestational diabetes and perinatal outcomes in Catalonia, Spain, 2006 to 2015: the diagestcat study. *Diabetes Metab Res Rev.* 2019;35(5): e3151. 10.1002/dmrr.3151 30865356

[4] Lavery J Friedman A Keyes K : Gestational diabetes in the United States: temporal changes in prevalence rates between 1979 and 2010. *BJOG.* 2017;124(5):804–13. 10.1111/1471-0528.14236 27510598 PMC5303559

[5] Johns EC Denison FC Norman JE : Gestational Diabetes Mellitus: mechanisms, treatment, and complications. *Trends Endocrinol Metab.* 2018;29(11):743–54. 10.1016/j.tem.2018.09.004 30297319

[6] Yang Y Wu N : Gestational Diabetes Mellitus and Preeclampsia: correlation and influencing factors. *Front Cardiovasc Med.* 2022;9: 831297. 10.3389/fcvm.2022.831297 35252402 PMC8889031

[7] Ye W Luo C Huang J : Gestational Diabetes Mellitus and adverse pregnancy outcomes: systematic review and meta-analysis. *BMJ.* 2022;377: e067946. 10.1136/bmj-2021-067946 35613728 PMC9131781

[8] Meregaglia M Dainelli L Banks H : The short-term economic burden of Gestational Diabetes Mellitus in Italy. *BMC Pregnancy Childbirth.* 2018;18(1): 58. 10.1186/s12884-018-1689-1 29471802 PMC5824573

[9] Sosa-Rubi SG Dainelli L Silva-Zolezzi I : Short-term health and economic burden of Gestational Diabetes Mellitus in Mexico: a modeling study. *Diabetes Res Clin Pract.* 2019;153:114–24. 10.1016/j.diabres.2019.05.014 31108135

[10] Xu T Dainelli L Yu K : The short-term health and economic burden of Gestational Diabetes Mellitus in China: a modelling study. *BMJ Open.* 2017;7(12): e018893. 10.1136/bmjopen-2017-018893 29203507 PMC5736026

[11] Kim C Berger DK Chamany S : Recurrence of Gestational Diabetes Mellitus: a systematic review. *Diabetes Care.* 2007;30(5):1314–9. 10.2337/dc06-2517 17290037

[12] Gadve SS Chavanda S Mukherjee AD : Risk of developing type 2 diabetes mellitus in South Asian women with history of Gestational Diabetes Mellitus: a systematic review and meta-analysis. *Indian J Endocrinol Metab.* 2021;25(3):176–81. 10.4103/ijem.IJEM_57_21 34760669 PMC8547406

[13] Vounzoulaki E Khunti K Abner SC : Progression to type 2 diabetes in women with a known history of gestational diabetes: systematic review and meta-analysis. *BMJ.* 2020;369: m1361. 10.1136/bmj.m1361 32404325 PMC7218708

[14] Cheung NW Byth K : Population health significance of gestational diabetes. *Diabetes Care.* 2003;26(7):2005–9. 10.2337/diacare.26.7.2005 12832303

[15] Xie W Wang Y Xiao S : Association of Gestational Diabetes Mellitus with overall and type specific cardiovascular and cerebrovascular diseases: systematic review and meta-analysis. *BMJ.* 2022;378: e070244. 10.1136/bmj-2022-070244 36130740 PMC9490552

[16] Li J Song C Li C : Increased risk of cardiovascular disease in women with prior gestational diabetes: a systematic review and meta-analysis. *Diabetes Res Clin Pract.* 2018;140:324–38. 10.1016/j.diabres.2018.03.054 29655653

[17] Foo RX Ma JJ Du R : Gestational Diabetes Mellitus and development of intergenerational Non-Alcoholic Fatty Liver Disease (NAFLD) after delivery: a systematic review and meta-analysis. *eClinicalMedicine.* 2024;72: 102609. 10.1016/j.eclinm.2024.102609 38707911 PMC11067479

[18] Tong GX Cheng J Chai J : Association between Gestational Diabetes Mellitus and subsequent risk of cancer: a systematic review of epidemiological studies. *Asian Pac J Cancer Prev.* 2014;15(10):4265–9. 10.7314/apjcp.2014.15.10.4265 24935382

[19] Barrett PM McCarthy FP Kublickiene K : Adverse pregnancy outcomes and long-term maternal kidney disease: a systematic review and meta-analysis. *JAMA Netw Open.* 2020;3(2): e1920964. 10.1001/jamanetworkopen.2019.20964 32049292 PMC12527481

[20] Flachs Madsen LR Gerdøe-Kristensen S Lauenborg J : Long-term follow-up on morbidity among women with a history of Gestational Diabetes Mellitus: a systematic review. *J Clin Endocrinol Metab.* 2022;107(9):2411–23. 10.1210/clinem/dgac373 35763540 PMC9387689

[21] Slouha E Gates KM Al-Geizi H : The relationship between gestational diabetes and the risk of cancer: a systematic review. *Cureus.* 2024;16(1): e53328. 10.7759/cureus.53328 38435884 PMC10906975

[22] Lowe WL Lowe LP Kuang A : Maternal glucose levels during pregnancy and childhood adiposity in the hyperglycemia and adverse pregnancy outcome follow-up study. *Diabetologia.* 2019;62(4):598–610. 10.1007/s00125-018-4809-6 30648193 PMC6421132

[23] Pathirana MM Lassi ZS Ali A : Association between metabolic syndrome and Gestational Diabetes Mellitus in women and their children: a systematic review and meta-analysis. *Endocrine.* 2021;71(2):310–20. 10.1007/s12020-020-02492-1 32930949

[24] Simmons D Gupta Y Hernandez TL : Call to action for a life course approach. *Lancet.* 2024;404(10448):193–214. 10.1016/S0140-6736(24)00826-2 38909623

[25] Fu J Retnakaran R : The life course perspective of gestational diabetes: an opportunity for the prevention of diabetes and heart disease in women. *eClinicalMedicine.* 2022;45: 101294. 10.1016/j.eclinm.2022.101294 35198924 PMC8850315

[26] Morton S Kirkwood S Thangaratinam S : Interventions to modify the progression to type 2 diabetes mellitus in women with gestational diabetes: a systematic review of literature. *Curr Opin Obstet Gynecol.* 2014;26(6):476–86. 10.1097/GCO.0000000000000127 25333677

[27] Lee VY Monjur MR Santos JA : The efficacy of interventions to prevent type 2 diabetes among women with recent Gestational Diabetes Mellitus—A living systematic review and meta-analysis. *J Diabetes.* 2024;16(8): e13590. 10.1111/1753-0407.13590 39136500 PMC11320752

[28] Retnakaran M Viana LV Kramer CK : Lifestyle intervention for the prevention of type 2 diabetes in women with prior gestational diabetes: a systematic review and meta-analysis. *Diabetes Obes Metab.* 2023;25(5):1196–202. 10.1111/dom.14966 36594235

[29] D’Arcy E Rayner J Hodge A : The role of diet in the prevention of diabetes among women with prior gestational diabetes: a systematic review of intervention and observational studies. *J Acad Nutr Diet.* 2020;120(1):69–85. e7. 10.1016/j.jand.2019.07.021 31636052

[30] Wang Y Wei W Guo H : Postpartum life interventions to prevent type 2 diabetes in women with gestational diabetes: a systematic review and meta-analysis. *J Diabetes Investig.* 2024;15(8):1115–28. 10.1111/jdi.14220 38727771 PMC11292388

[31] Guo J Chen JL Whittemore R : Postpartum lifestyle interventions to prevent type 2 diabetes among women with history of gestational diabetes: a systematic review of randomized clinical trials. *J Womens Health (Larchmt).* 2016;25(1):38–49. 10.1089/jwh.2015.5262 26700931

[32] Goveia P Cañon-Montañez W Santos DP : Lifestyle intervention for the prevention of diabetes in women with previous Gestational Diabetes Mellitus: a systematic review and meta-analysis. *Front Endocrinol (Lausanne).* 2018;9:583. 10.3389/fendo.2018.00583 30344509 PMC6182069

[33] Peacock AS Bogossian F McIntyre HD : A review of interventions to prevent type 2 diabetes after gestational diabetes. *Women Birth.* 2014;27(4):e7–15. 10.1016/j.wombi.2014.09.002 25262356

[34] Lie ML Hayes L Lewis-Barned NJ : Preventing type 2 diabetes after gestational diabetes: women’s experiences and implications for diabetes prevention interventions. *Diabet Med.* 2013;30(8):986–93. 10.1111/dme.12206 23534548

[35] Cheung NW Blumenthal C Smith BJ : A pilot randomised controlled trial of a text messaging intervention with customisation using linked data from wireless wearable activity monitors to improve risk factors following gestational diabetes. *Nutrients.* 2019;11(3):590. 10.3390/nu11030590 30862052 PMC6470941

[36] O’Reilly SL Dunbar JA Versace V : Mothers After Gestational Diabetes in Australia (MAGDA): a randomised controlled trial of a postnatal diabetes prevention program. *PLoS Med.* 2016;13(7): e1002092. 10.1371/journal.pmed.1002092 27459502 PMC4961439

[37] Holmes VA Draffin CR Patterson CC : Postnatal lifestyle intervention for overweight women with previous gestational diabetes: a randomized controlled trial. *J Clin Endocrinol Metab.* 2018;103(7):2478–87. 10.1210/jc.2017-02654 29762737

[38] Zilberman-Kravits D Meyerstein N Abu-Rabia Y : The impact of a cultural lifestyle intervention on metabolic parameters after Gestational Diabetes Mellitus a randomized controlled trial. *Matern Child Health J.* 2018;22(6):803–11. 10.1007/s10995-018-2450-0 29411251

[39] O’Dea A Tierney M McGuire BE : Can the onset of type 2 diabetes be delayed by a group-based lifestyle intervention in women with prediabetes following Gestational Diabetes Mellitus (GDM)? Findings from a randomized control mixed methods trial. *J Diabetes Res.* 2015;2015(1): 798460. 10.1155/2015/798460 26347894 PMC4546980

[40] Dennison RA Oliver-Williams C Qi HLJ : The effectiveness of pharmacological and lifestyle interventions to reduce the risk of diabetes and hyperglycaemia following gestational diabetes: a systematic review and meta-analysis. *Diabet Med.* 2024;41(6): e15316. 10.1111/dme.15316 38553834

[41] Recommendations: Diabetes in pregnancy: management from preconception to the postnatal period.Guidance, NICE,2015; [cited 2024 Sep 9]. Reference Source

[42] Ukke GG Boyle JA Reja A : Lifestyle interventions to prevent type 2 Diabetes in women with a history of gestational diabetes: a systematic review and meta-analysis through the lens of health equity. *Nutrients.* 2023;15(21):4666. 10.3390/nu15214666 37960319 PMC10649749

[43] Werbrouck A Schmidt M Putman K : A systematic review on costs and cost-effectiveness of screening and prevention of type 2 diabetes in women with prior gestational diabetes: exploring uncharted territory. *Diabetes Res Clin Pract.* 2019;147:138–148. 10.1016/j.diabres.2018.11.012 30529576

[44] Li W Kim CS Howell EA : Economic evaluation of prenatal and postpartum care in women with gestational diabetes and Hypertensive Disorders of Pregnancy: a systematic review. *Value Health.* 2022;25(12):2062–2080. 10.1016/j.jval.2022.07.014 35989155 PMC9669139

[45] Phelan S Jelalian E Coustan D : Randomized controlled trial of prepregnancy lifestyle intervention to reduce recurrence of Gestational Diabetes Mellitus. *Am J Obstet Gynecol.* 2023;229(2):158.e1–158. e14. 10.1016/j.ajog.2023.01.037 36758710

[46] Quotah OF Andreeva D Nowak KG : Interventions in preconception and pregnant women at risk of gestational diabetes; a systematic review and meta-analysis of Randomised Controlled Trials. *Diabetol Metab Syndr.* 2024;16(1): 8. 10.1186/s13098-023-01217-4 38178175 PMC10765912

[47] Tieu J Shepherd E Middleton P : Interconception care for women with a history of gestational diabetes for improving maternal and infant outcomes. *Cochrane Database Syst Rev.* Cochrane Library,2017;8(8): CD010211. 10.1002/14651858.CD010211.pub3 28836274 PMC6483533

[48] Ohene-Agyei P Iqbal A Harding JE : Postnatal care after gestational diabetes – a systematic review of clinical practice guidelines. *BMC Pregnancy Childbirth.* 2024;24(1): 720. 10.1186/s12884-024-06899-w 39497079 PMC11536828

[49] Fiskå BS Pay ASD Staff AC : Gestational Diabetes Mellitus, follow-up of future maternal risk of cardiovascular disease and the use of eHealth technologies—a scoping review. *Syst Rev.* 2023;12(1): 178. 10.1186/s13643-023-02343-w 37770980 PMC10537141

[50] Hod M Kapur A Sacks DA : The International Federation of Gynecology and Obstetrics (FIGO) initiative on Gestational Diabetes Mellitus: a pragmatic guide for diagnosis, management, and care. *Int J Gynecol Obstet.* 2015;131 Suppl(S3):S173–211. 10.1016/S0020-7292(15)30033-3 26433807

[51] Diabetes Canada Clinical Practice Guidelines Expert Committee: Feig DS Berger H : Diabetes and pregnancy. *Can J Diabetes.* 2018;42 Suppl 1:S255–S282. 10.1016/j.jcjd.2017.10.038 29650105

[52] American Diabetes Association Professional Practice Committee: 15. Management of diabetes in pregnancy: *Standards of Care in Diabetes- 2024*. *Diabetes Care.* 2024;47(Suppl 1):S282–S294. 10.2337/dc24-S015 38078583 PMC10725801

[53] Smirnakis KV Chasan-Taber L Wolf M : Postpartum diabetes screening in women with a history of gestational diabetes. *Obstet Gynecol.* 2005;106(6):1297–303. 10.1097/01.AOG.0000189081.46925.90 16319255

[54] Bernstein JA Quinn E Ameli O : Follow-up after gestational diabetes: a fixable gap in women’s preventive healthcare. *BMJ Open Diabetes Res Care.* 2017;5(1): e000445. 10.1136/bmjdrc-2017-000445 28948028 PMC5595177

[55] Chittleborough CR Baldock KL Taylor AW : Long-term follow-up of women with Gestational Diabetes Mellitus: the South Australian Gestational Diabetes Mellitus Recall Register. *Aust N Z J Obstet Gynaecol.* 2010;50(2):127–31. 10.1111/j.1479-828X.2010.01140.x 20522067

[56] Wilkinson SA Lim SS Upham S : Who’s responsible for the care of women during and after a pregnancy affected by gestational diabetes? *Med J Aust.* 2014;201(3 Suppl):S78–81. 10.5694/mja14.00251 25047889

[57] Valabhji J Barron E Bradley D : Early outcomes from the English National Health Service Diabetes Prevention Programme. *Diabetes Care.* 2020;43(1):152–60. 10.2337/dc19-1425 31719054 PMC7115827

[58] Dennison RA Ward RJ Griffin SJ : Women’s views on lifestyle changes to reduce the risk of developing type 2 diabetes after gestational diabetes: a systematic review, qualitative synthesis and recommendations for practice. *Diabet Med.* 2019;36(6):702–17. 10.1111/dme.13926 30723968 PMC6563496

[59] Keely E Clark H Karovitch A : Screening for type 2 diabetes following gestational diabetes. *Can Fam Physician.* 2010;56(6):558–63. 20547525 PMC2902390

[60] Ehrenthal DB Maiden K Rogers S : Postpartum healthcare after gestational diabetes and hypertension. *J Womens Health (Larchmt).* 2014;23(9):760–4. 10.1089/jwh.2013.4688 25089915 PMC4158990

[61] Lithgow GE Rossi J Griffin SJ : Barriers to postpartum diabetes screening: a qualitative synthesis of clinicians’ views. *Br J Gen Pract.* 2021;71(707):e473–e482. 10.3399/BJGP.2020.0928 33947667 PMC8103924

[62] Shea AK Shah BR Clark HD : The effectiveness of implementing a reminder system into routine clinical practice: does it increase postpartum screening in women with gestational diabetes? *Chronic Dis Can.* 2011;31(2):58–64. 10.24095/hpcdp.31.2.02 21466755

[63] Arksey H O’Malley L : Scoping studies: towards a methodological framework. *Int J Soc Res Methodol.* 2005;8(1):19–32. 10.1080/1364557032000119616

[64] Peters MD Godfrey C McInerney P : Scoping reviews.In: Aromataris E, Lockwood C, Porritt K, Pilla B, Jordan Z, editors. *JBI Manual for Evidence Synthesis*. JBI,2024. 10.46658/JBIMES-24-09

[65] Shamseer L Moher D Clarke M : Preferred Reporting Items for Systematic review and Meta-Analysis Protocols (PRISMA-P) 2015: elaboration and explanation. *BMJ.* 2015;350: g7647. 10.1136/bmj.g7647 25555855

[66] Li-zhen L Yun X Xiao-Dong Z : Evaluation of guidelines on the screening and diagnosis of Gestational Diabetes Mellitus: systematic review. *BMJ Open.* 2019;9(5): e023014. 10.1136/bmjopen-2018-023014 31061012 PMC6502228

[67] Definition of General Practice/Family Medicine. WONCA Europe. [cited 2024 Sep 9]. Reference Source

[68] Jackson JL Kuriyama A Anton A : The accuracy of Google Translate for abstracting data from non-English-language trials for systematic reviews. *Ann Intern Med.* 2019;171(9):677–679. 10.7326/M19-0891 31357212

[69] Popel M Tomkova M Tomek J : Transforming machine translation: a deep learning system reaches news translation quality comparable to human professionals. *Nat Commun.* 2020;11(1): 4381. 10.1038/s41467-020-18073-9 32873773 PMC7463233

[70] HAPO Study Cooperative Research Group, Metzger BE Lowe LP : Hyperglycemia and adverse pregnancy outcomes. *N Engl J Med.* 2008;358(19):1991–2002. 10.1056/NEJMoa0707943 18463375

[71] Tricco AC Lillie E Zarin W : PRISMA extension for Scoping Reviews (PRISMA-ScR): checklist and explanation. *Ann Intern Med.* 2018;169(7):467–473. 10.7326/M18-0850 30178033

[72] Levac D Colquhoun H O’Brien KK : Scoping studies: advancing the methodology. *Implement Sci.* 2010;5: 69. 10.1186/1748-5908-5-69 20854677 PMC2954944

[73] Tugwell P Petticrew M Robinson V : Cochrane and Campbell Collaborations, and health equity. *Lancet.* 2006;367(9517):1128–30. 10.1016/S0140-6736(06)68490-0 16616547

[74] O’Neill J Tabish H Welch V : Applying an equity lens to interventions: using PROGRESS ensures consideration of socially stratifying factors to illuminate inequities in health. *J Clin Epidemiol.* 2014;67(1):56–64. 10.1016/j.jclinepi.2013.08.005 24189091

[75] Petticrew M Whitehead M Macintyre SJ : Evidence for public health policy on inequalities: 1: the reality according to policymakers. *J Epidemiol Community Health.* 2004;58(10):811–6. 10.1136/jech.2003.015289 15365104 PMC1763325

[76] Hoffmann TC Glasziou PP Boutron I : Better reporting of interventions: Template for Intervention Description and Replication (TIDieR) checklist and guide. *BMJ.* 2014;348: g1687. 10.1136/bmj.g1687 24609605

[77] Wu N O’Reilly S Nielsen KK : Core outcome set for diabetes after pregnancy prevention across the life span: international Delphi study. *BMJ Open Diabetes Res Care.* 2020;8(2): e001594. 10.1136/bmjdrc-2020-001594 33148689 PMC7640499

[78] Bogdanet D Reddin C Macken E : Follow-up at 1 year and beyond of women with Gestational Diabetes treated with insulin and/or oral glucose-lowering agents: a core outcome set using a Delphi survey. *Diabetologia.* 2019;62(11):2007–2016. 10.1007/s00125-019-4935-9 31273408 PMC6805965

[79] Pope C Ziebland S Mays N : Analysing qualitative data. *BMJ.* 2000;320(7227):114–116. 10.1136/bmj.320.7227.114 10625273 PMC1117368

[80] Ritchie J Spencer L : Qualitative data analysis for applied policy research.In: Bryman A, Burgess RG, editors. *Analyzing qualitative data.*Abingdon, UK: Taylor & Francis,1994;173–94. Reference Source

[81] Carroll C Booth A Leaviss J : “Best fit” framework synthesis: refining the method. *BMC Med Res Methodol.* 2013;13(1): 37. 10.1186/1471-2288-13-37 23497061 PMC3618126

[82] Carroll C Booth A Cooper K : A worked example of ‘best fit’ framework synthesis: a systematic review of views concerning the taking of some potential chemopreventive agents. *BMC Med Res Methodol.* 2011;11(1): 29. 10.1186/1471-2288-11-29 21410933 PMC3068987

[83] Wagner EH : Chronic disease management: what will it take to improve care for chronic illness? *Eff Clin Pract.* 1998;1(1):2–4. 10345255

[84] Bodenheimer T Wagner EH Grumbach K : Improving primary care for patients with chronic illness. *JAMA.* 2002;288(14):1775–9. 10.1001/jama.288.14.1775 12365965

[85] Bodenheimer T Wagner EH Grumbach K : Improving primary care for patients with chronic illness: the chronic care model, Part 2. *JAMA.* 2002;288(15):1909–14. 10.1001/jama.288.15.1909 12377092

[86] Tsai AC Morton SC Mangione CM : A meta-analysis of interventions to improve care for chronic illnesses. *Am J Manag Care.* 2005;11(8):478–88. 16095434 PMC3244301

[87] Vargas RB Mangione CM Asch S : Can a chronic care model collaborative reduce heart disease risk in patients with diabetes? *J Gen Intern Med.* 2007;22(2):215–22. 10.1007/s11606-006-0072-5 17356989 PMC1824758

[88] Davy C Bleasel J Liu H : Effectiveness of chronic care models: opportunities for improving healthcare practice and health outcomes: a systematic review. *BMC Health Serv Res.* 2015;15: 194. 10.1186/s12913-015-0854-8 25958128 PMC4448852

[89] Goh LH Siah CJR Tam WWS : Effectiveness of the chronic care model for adults with type 2 diabetes in primary care: a systematic review and meta-analysis. *Syst Rev.* 2022;11(1): 273. 10.1186/s13643-022-02117-w 36522687 PMC9753411

[90] American Diabetes Association Professional Practice Committee: 1. Improving care and promoting health in populations: *standards of medical care in diabetes— 2022.* *Diabetes Care.* 2022;45(Supplement_ 1):S8–16. 10.2337/dc22-S001 34964872

[91] Miles MB Huberman AM : Qualitative data analysis: an expanded sourcebook.2nd ed. Thousand Oaks: Sage Publications,1994; 338. Reference Source

[92] Wong EC Maher AR Motala A : Methods for identifying health research gaps, needs, and priorities: a scoping review. *J Gen Intern Med.* 2022;37(1):198–205. 10.1007/s11606-021-07064-1 34748098 PMC8738821

[93] Robinson KA Saldanha IJ McKoy NA : Development of a framework to identify research gaps from systematic reviews. *J Clin Epidemiol.* 2011;64(12):1325–30. 10.1016/j.jclinepi.2011.06.009 21937195

[94] Saldanha IJ Wilson LM Bennett WL : Development and pilot test of a process to identify research needs from a systematic review. *J Clin Epidemiol.* 2013;66(5):538–45. 10.1016/j.jclinepi.2012.07.009 22995855

