# Supplementary material for: The long-term general practice healthcare of women with a history of gestational diabetes: A Scoping Review Protocol
Source: HRB Open Res. 2025 Apr 10;8:31. Originally published 2025 Feb 11. [Version 2] doi: 10.12688/hrbopenres.14022.2 (PMC11992512; doi:10.12688/hrbopenres.14022.2)
Supplement: Peer review history

# Reviewer report: Reviewer response for version 2

**DOI:** 10.21956/hrbopenres.15532.r46733

**Published:** 2025-04-11

**Authors:** Alpesh Goyal

**Affiliations:** 1 Department of Endocrinology and Metabolism, All India Institute of Medical Sciences, Bhopal, Madhya Pradesh, India

Thanks for revising the manuscript in line with the reviewer suggestions. The changes incorporated are appropriate and improve the quality of this paper. I have no further comments.

Is the study design appropriate for the research question?

Yes

Is the rationale for, and objectives of, the study clearly described?

Yes

Are sufficient details of the methods provided to allow replication by others?

Yes

Are the datasets clearly presented in a useable and accessible format?

Not applicable

Reviewer Expertise:

Diabetes in Pregnancy and Adrenal disorders

I confirm that I have read this submission and believe that I have an appropriate level of expertise to confirm that it is of an acceptable scientific standard.

# Reviewer report: Reviewer response for version 1

**DOI:** 10.21956/hrbopenres.15392.r46023

**Published:** 2025-03-07

**Authors:** Alpesh Goyal

**Affiliations:** 1 Department of Endocrinology and Metabolism, All India Institute of Medical Sciences, Bhopal, Madhya Pradesh, India

Thanks for the opportunity to review. The authors aim to perform a scoping review about long-term care of women with GDM in healthcare practices and the research gaps therein. The authors present a protocol for this review, which is fairly well written. I have the following comments/suggestions:

 1. Background para 2: there are updated studies that highlight the link between GDM and steatosis and may be referred to (Kubihal S, et al., 2021 [Ref 1] and Foo RX, et al., 2024 [Ref 2]).

 2. Background para 3: Authors state that lifestyle interventions are efficacious to prevent T2DM after GDM. However, I would like to draw their attention to the most updated meta-analysis on efficacy of interventions to prevent T2DM after GDM by Lee et al (Journal of Diabetes 2024; Lee VY, et al., 2024 [Ref 3]). This MA reported equivocal efficacy, and highlighted the need for more pharmacology trials in this field. Thus, the evidence, at this time, is equivocal and more research is needed, especially the role of pharmacological interventions. The MA by Lee et al focused on individuals with GDM complicated pregnancy within 5 years postpartum, and hence excluded the DPP GDM subgroup (mean 656 weeks postpartum).  Please account for these recent updates in your description.

 3. I agree that the postpartum screening rates are quite dismal and reported at <50% in most studies (Keely E 2024 [Ref 4], Goyal A, et al., 2018 [Ref 5].) Is that also a thrust area for your scoping review?

 4. What time window will be used to define "long-term postpartum follow-up" or "beyond initial postpartum screening"?. In many settings, the initial postpartum screen may not happen at 6-12 weeks and be delayed for years.

Is the study design appropriate for the research question?

Yes

Is the rationale for, and objectives of, the study clearly described?

Yes

Are sufficient details of the methods provided to allow replication by others?

Yes

Are the datasets clearly presented in a useable and accessible format?

Not applicable

Reviewer Expertise:

Diabetes in Pregnancy and Adrenal disorders

I confirm that I have read this submission and believe that I have an appropriate level of expertise to confirm that it is of an acceptable scientific standard, however I have significant reservations, as outlined above.

## References

[1] : Prevalence of non-alcoholic fatty liver disease and factors associated with it in Indian women with a history of gestational diabetes mellitus. *J Diabetes Investig* .2021;12(5) : 10.1111/jdi.13411 877-885 10.1111/jdi.13411 32961610 PMC8089012

[2] : Gestational diabetes mellitus and development of intergenerational non-alcoholic fatty liver disease (NAFLD) after delivery: a systematic review and meta-analysis. *EClinicalMedicine* .2024;72: 10.1016/j.eclinm.2024.102609 102609 10.1016/j.eclinm.2024.102609 38707911 PMC11067479

[3] : The efficacy of interventions to prevent type 2 diabetes among women with recent gestational diabetes mellitus-A living systematic review and meta-analysis. *J Diabetes* .2024;16(8) : 10.1111/1753-0407.13590 e13590 10.1111/1753-0407.13590 39136500 PMC11320752

[4] : An opportunity not to be missed--how do we improve postpartum screening rates for women with gestational diabetes?. *Diabetes Metab Res Rev* .2012;28(4) : 10.1002/dmrr.2274 312-6 10.1002/dmrr.2274 22228674

[5] : Long term (>1 year) postpartum glucose tolerance status among Indian women with history of Gestational Diabetes Mellitus (GDM) diagnosed by IADPSG criteria. *Diabetes Res Clin Pract* .2018;142: 10.1016/j.diabres.2018.05.027 154-161 10.1016/j.diabres.2018.05.027 29802954

# Response

**Published:** 2025-03-31

**Authors:** James O'Flynn

**Affiliations:** Department of General Practice, University College Cork School of Medicine, Cork, County Cork, Ireland

We are most grateful to the reviewer for this detailed contribution. We have submitted an updated version of the protocol that takes into consideration the points made by the reviewer, and which we feel is stronger as a result. We would like to take the opportunity to submit some more specific responses to the points made by the reviewer here:

- The link between hepatic steatosis
  - Thank you for signposting towards these updated studies. We have swapped in the larger and more recent of your two suggestions (the review by Foo and colleagues).
- The efficacy of lifestyle interventions in preventing Type 2 Diabetes after GDM (Background, paragraph 3)
  - Thank you for this comment. Indeed, statistical significance of the effects of lifestyle and pharmacological interventions on the incidence of type 2 diabetes and other outcome measures is inconsistent across the literature. The analysis you cite may raise the possibility of intervention and outcome timing as important factors. We have edited the text to emphasise the statistical limitations of the current literature on the subject: "A recent meta-analysis found that the hazard ratio of type 2 diabetes was significantly improved by interventions, while the relative risk did not achieve significance.1 This may occur if the effect of interventions is time-dependent, and may suggest that their effect may more readily delay than outright prevent the onset of type 2 diabetes." Thank you.
- Postpartum Screening as a thrust area for the review
  - Thank you for this comment. Indeed the rates are disappointing and this is indeed a key reason why we are performing this research. We have re-emphasised this in the last paragraph of the introduction: "Therefore, a comprehensive understanding of long-term general practice healthcare for women with a history of GDM is required *if the rates and quality of follow-up are to be improved*."
- The time window for "long-term postpartum follow-up"
  - Thank you for raising this point. We had considered defining a strict minimum time frame for inclusion, and ultimately decided that a more flexible definition would be appropriate. We agree with your point that there is variability in the occurrence of that initial test, with the recommendations for the initial follow-up screening to be between 4 weeks at the earliest, to 6 months in some guidelines, and sometimes substantially longer in practicality.2 In addition, some study types (for example, qualitative studies of women’s experiences of healthcare) may explore a continuous timeframe from antenatal to years later. Because of these factors, a strict time-frame may impair the selection of some highly relevant papers. Authors’ judgments will be used to interpret whether candidate papers focus only on the initial postpartum period or contain data looking beyond that. To reduce confusion, we have clarified the text to state that we want to explore research that explores beyond the early post-partum “period” rather than healthcare after initial “screening”. Thank you.

The review provided valuable opportunities to improve the accuracy and clarity of the protocol, and we feel that the newly submitted version has benefitted from these changes. Thank you.

 References:

 1. Lee VY, Monjur MR, Santos JA, Patel A, Liu R, Di Tanna GL, et al. The efficacy of interventions to prevent type 2 diabetes among women with recent gestational diabetes mellitus—A living systematic review and meta-analysis. J Diabetes. 2024;16(8):e13590. 

 2. Ohene-Agyei P, Iqbal A, Harding JE, Crowther CA, Lin L. Postnatal care after gestational diabetes – a systematic review of clinical practice guidelines. BMC Pregnancy Childbirth. 2024 Nov 4;24(1):720.

# Reviewer report: Reviewer response for version 1

**DOI:** 10.21956/hrbopenres.15392.r45820

**Published:** 2025-03-06

**Authors:** Angela Flynn

**Affiliations:** 1 Royal College of Surgeons in Ireland, Dublin, Ireland

Overall, this scoping review protocol is very well written, and focuses on the important topic of optimising health and reducing the risk of chronic disease in women with previous GDM. The protocol outlines the methodology that will be undertaken to map the existing research evidence on longterm GP based healthcare for women with previous GDM.

 My major comment is increasing the clarity of the ‘concept’ of the scoping review. It needs to be clear that this review is mapping the research evidence and is not a review of current healthcare practices. The concept in Table 1 needs to reflect this as the currently stated ‘characteristics of healthcare’ does not suggest a research focus. Ensure it is clear throughout the article that the review is mapping research evidence related to the longterm care of women with previous GDM and is not evaluating health services.

 Other comments:

 It is not clear from the background what is meant by ‘longterm care’. What do the authors mean by ‘longterm healthcare’ for women with previous GDM?

 It is worth noting in the background although primary care has been identified as an appropriate setting for longterm follow up, there are also several prevention programmes rolled out internationally to prevent the risk of chronic disease in women with previous GDM. Diabetes prevention programmes exist in several countries and are delivered virtually, either through self referral or referral through primary care. GPs have a role to ensure women can access existing programmes.

 The background is focused on prevention of chronic disease, however, the concept also focuses on prevention of GDM recurrence. Optimising preconception health in women with previous GDM is part of longterm care but is not mentioned in the background. There are multiple research papers on preconception care and how preconception care can be implemented in primary care; Schoenaker D, et al., 2024 (Ref 1) and Schoenaker D, et al., 2022 (Ref 2)

 Due to the nature of the research, the authors should include adding in a grey literature search. It might be useful to identify educational interventions in primary care settings.

 Agree with ensuring the search strategy is relevant, however, what is the rationale for the search being limited to the last 10 years? Please add in.

Is the study design appropriate for the research question?

Yes

Is the rationale for, and objectives of, the study clearly described?

Yes

Are sufficient details of the methods provided to allow replication by others?

Yes

Are the datasets clearly presented in a useable and accessible format?

Not applicable

Reviewer Expertise:

Maternal health research, in particular preconception, pregnancy, and postpartum research of women at high metabolic risk eg women living with obesity or those who develop diabetes in pregnancy

I confirm that I have read this submission and believe that I have an appropriate level of expertise to confirm that it is of an acceptable scientific standard.

## References

[1] : Developing consensus on priorities for preconception care in the general practice setting in the UK: Study protocol. *PLoS One* .2024;19(11) : 10.1371/journal.pone.0311578 e0311578 10.1371/journal.pone.0311578 39570956 PMC11581211

[2] : Preconception care in primary care: supporting patients to have healthier pregnancies and babies. *Br J Gen Pract* .2022;72(717) : 10.3399/bjgp22X718853 152 10.3399/bjgp22X718853 35361583 PMC8966926

# Response

**Published:** 2025-03-31

**Authors:** James O'Flynn

**Affiliations:** Department of General Practice, University College Cork School of Medicine, Cork, County Cork, Ireland

We are very thankful for this peer review. We have considered the feedback and submitted an updated version of the protocol. We would like to take this opportunity to more directly respond to the points made by the reviewer:

- Mapping Evidence rather than evaluating health services
  - Thank you for raising this point. We have made adjustments emphasising that our focus is to review the research rather than to evaluate health services. These changes occur in the Objectives, in Table 1, in the first line of the Concept paragraph, in the Discussion and in the Conclusion. Thank you.
- The meaning of "Long term care"
  - Thank you for identifying this opportunity to improve the clarity of this concept. We have edited the fourth paragraph of the introduction to say that “this may include lifelong regular screening for early diagnosis of diabetes or cardiovascular disease, and ongoing support for maintenance of positive health behaviours”. We would hope that this gives clarity on the timeframe and the broad content of this care.
- Prevention programmes including DPP
  - Thank you. We agree with your suggestion that such programmes are sufficiently important to necessitate their inclusion in this protocol, and have now done so in the introduction: "GPs can provide direct support and can connect women with parallel services, including Diabetes Prevention Programmes."1,2 However, a detailed discussion of diabetes prevention programmes is omitted in this general-practice focused review.
- The importance of preventing GDM recurrence
  - Thank you for this comment. We agree that the protocol may benefit from emphasis on this important risk for these women. We have included a point on this within the introduction: "Data regarding the role for preconception care aimed at reducing the risk of GDM recurrence are also lacking.3,4,5. In spite of this, clinical practice guidelines frequently identify the importance of family planning and contraception for women with a history of GDM.6" Thank you.
- Grey Literature
  - Thank you for raising this point. Grey literature may contain valuable sources, and, as you point out, may be particularly valuable for identifying educational interventions. However, the primary aim for this scoping review will be to synthesise empirical evidence from peer-reviewed publications. In the absence of an assessment of quality within a scoping review, this creates some standardisation of quality across the included studies. Furthermore, given the scope of the subject area, including grey literature would significantly expand the search and screening process, which may compromise the feasibility of this review. To improve the clarity of our protocol, we have stated our intention to omit grey literature more explicitly. Thank you.
- 10 year eligibility limit
  - Thank you for this comment. Rationale clarified within the text: "To accommodate the rapidly evolving healthcare landscape, and to permit permeation of the practice-changing findings of the Hyperglycemia and Adverse Pregnancy Outcomes study 2008, the search years will be limited to the past 10 years (2014 or later)"7

Again, we are most grateful for your review, which identifies a number of opportunities to strengthen this protocol. Thank you. 

 References:

 1. Valabhji J, Barron E, Bradley D, Bakhai C, Fagg J, O’Neill S, et al. Early Outcomes From the English National Health Service Diabetes Prevention Programme. Diabetes Care. 2019 Dec 12;43(1):152–60. 

 2. Dennison RA, Ward RJ, Griffin SJ, Usher‐Smith JA. Women’s views on lifestyle changes to reduce the risk of developing Type 2 diabetes after gestational diabetes: a systematic review, qualitative synthesis and recommendations for practice. Diabet Med. 2019 Jun;36(6):702–17. 

 3.    Phelan S, Jelalian E, Coustan D, Caughey AB, Castorino K, Hagobian T, et al. Randomized controlled trial of prepregnancy lifestyle intervention to reduce recurrence of gestational diabetes mellitus. Am J Obstet Gynecol. 2023 Aug 1;229(2):158.e1-158.e14. 

 4.    Quotah OF, Andreeva D, Nowak KG, Dalrymple KV, Almubarak A, Patel A, et al. Interventions in preconception and pregnant women at risk of gestational diabetes; a systematic review and meta-analysis of randomised controlled trials. Diabetol Metab Syndr. 2024 Jan 4;16:8. 

 5.    Tieu J, Shepherd E, Middleton P, Crowther CA. Interconception care for women with a history of gestational diabetes for improving maternal and infant outcomes - Tieu, J - 2017 | Cochrane Library. [cited 2025 Mar 20]; Available from: https://www.cochranelibrary.com/cdsr/doi/10.1002/14651858.CD010211.pub3/full?cookiesEnabled

 6.    Ohene-Agyei P, Iqbal A, Harding JE, Crowther CA, Lin L. Postnatal care after gestational diabetes – a systematic review of clinical practice guidelines. BMC Pregnancy Childbirth. 2024 Nov 4;24(1):720. 

 7.     HAPO Study Cooperative Research Group, Metzger BE, Lowe LP, Dyer AR, Trimble ER, Chaovarindr U, et al. Hyperglycemia and adverse pregnancy outcomes. N Engl J Med. 2008 May 8;358(19):1991–2002.
